# Modified conditioning regimen with idarubicin followed by autologous hematopoietic stem cell transplantation for invasive B-cell non-Hodgkin’s lymphoma patients

**DOI:** 10.1038/s41598-021-81944-8

**Published:** 2021-02-19

**Authors:** Chen Tian, Yueyang Li, Su Liu, Zehui Chen, Yizhuo Zhang, Yong Yu, Hongliang Yang, Haifeng Zhao, Zhigang Zhao, Tian Yuan, Yafei Wang

**Affiliations:** Key Laboratory of Cancer Prevention and Therapy, Department of Hematology, National Clinical Research Center for Cancer, Tianjin Medical University Cancer Institute and Hospital, Tianjin, 300060 China

**Keywords:** B-cell lymphoma, Haematopoietic stem cells

## Abstract

High-dose chemotherapy followed by autologous hematopoietic stem cell transplantation (ASCT) is still a consolidation treatment choice for relapsed/refractory B-cell non-Hodgkin’s lymphoma (NHL) patients and some aggressive B-cell NHL as frontline therapy. Due to the shortage of carmustine, we switched to idarubicin-substituted BEAC (IEAC) conditioning regimen. We retrospectively compared the outcomes of 72 aggressive B-cell NHL patients treated with IEAC or BEAC regimens followed by ASCT as upfront consolidative treatment. The median time to neutrophil and platelet reconstitution showed no difference between IEAC and BEAC groups. IEAC regimen was well tolerated without increase of adverse events. Transplant-related mortality didn’t occur. The overall survival (OS) and progression-free survival (PFS) of IEAC group (33 and 23 months) were a little longer than that of BEAC group (30 and 18 months). However, due to the small sample numbers, there’s no significant difference in OS and PFS between IEAC and BEAC group with DLBCL or MCL. Multivariate analysis showed that AnnArbor staging, IPI score, lactate dehydrogenase level, remission of disease, modified regimen were related with PFS and OS. In conclusion, IEAC regimen was well tolerated and replacement with idarubicin could be an alternative when carmustine was not available.

## Introduction

Non-Hodgkin's lymphoma (NHL) is the most common hematologic malignancy^1^. High-dose chemotherapy (HDC) has been a standard front-line therapy for patients with aggressive NHL for decades. Autologous hematopoietic stem cell transplantation (ASCT) is standard of care (SOC) for relapsed/refractory DLBCL^2–4^, which can eliminate the residual tumor cells, thereby decrease the probability of disease recurrence and prolong the survival^5^. BEAM (carmustine, etoposide, cytarabine, and melphalan), BEAC (carmustine, etoposide, cytarabine, cyclophosphamide) and CBV (carmustine, cyclophosphamide, and etoposide) are the most commonly used conditioning regimens for NHL^6,7^.

With the aim of obtaining a higher anti-lymphoma activity and/or reducing the toxic effects, a number of studies suggested the possibility of improving the outcomes of NHL patients through modifying the conditioning regimens^8–10^. BuCyE (busulfan, cyclophosphamide, and etoposide)^11,12^ and Benda-EAM (bendamustine, etoposide, cytarabine, and melphalan)^13,14^ were approved to be effective and safe for NHL patients^15,16^. However, idarubicin, which was a widely used anthracycline drug for NHL patients, was rarely reported to be added in conditioning regimen. In 1997, Engert et al. found that IIVP (ifosfamide, idarubicin, and etoposide) was a salvage regimen with acceptable toxicity and highly effective for patients with R/R NHL^17^. Due to the shortage of carmustine, bendamustine and nimustine in China, we aimed to examine conditioning with idarubicin and to compare the efficacy and toxicity between BEAC and idarubicin-substituted BEAC (IEAC).

## Methods

### Patients

**Figure 1:**
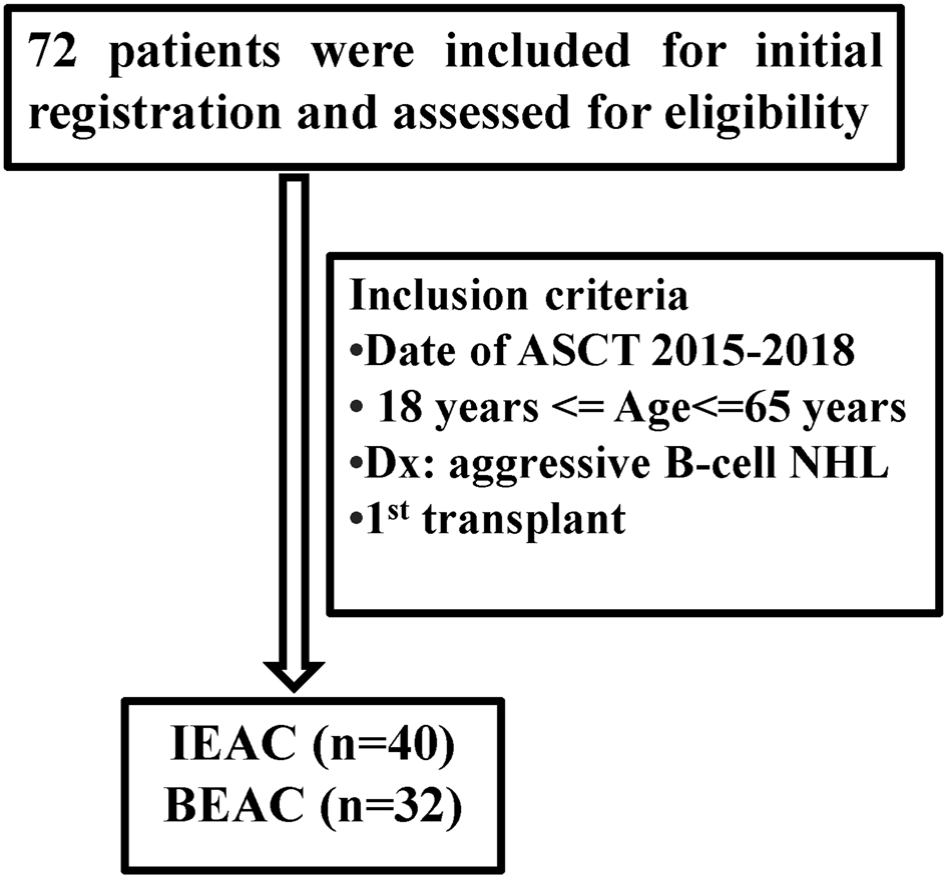
Study design.

This study was subject to approval by the Research Ethics Committee of Tianjin Medical University Cancer Institute and Hospital. All methods were carried out in accordance with relevant guidelines and regulations. Informed consent was obtained from all subjects. A retrospective study of 72 invasive B-cell NHL patients (18–65 years old) who received either IEAC (n = 40) or BEAC (n = 32) between 01/2015 and 06/2018 were enrolled, as shown in Fig. 1. All patients received 4–6 cycles of standard chemotherapy such as R-CHOP (doxorubicin 50 mg/m^2^) or R-DA-EPOCH (doxorubicin 10 mg/m^2^/day infusion × 96 h, days 1–4) regimen and performed ^18^-fluorodeoxyglucose positron emission tomography/computed tomography (PET–CT) to evaluate remission state before and after ASCT (Fig. 1). The invasive B cell NHL patients included diffuse large B cell lymphoma (DLBCL) and mantle cell lymphoma (MCL) patients in first CR with high-risk factors and patients in PR after 6 cycles of standard induction therapy. High-risk factors included central nervous system infiltration (primary and secondary), high grade B cell lymphoma (exclude Burkitt and double/triple hit lymphoma), international prognostic index (IPI) score > 3.

### Treatment protocols

At transplantation, patients were expected to have a Karnofsky performance status > 80, together with adequate cardiac (EJF > or equal to 50%), liver, and lung function, and no infectious process. Patients were treated with either BEAC regimen consisting of Carmustine (BCNU, 300 mg/m^2^ on day − 6), Etoposide (100 mg/m^2^ every 12 h on days − 5 to − 2), Cytarabine (200 mg/m^2^ every 12 h on days − 5 to − 2), and Cyclophosphamide (1.5 g/m^2^ on days − 5 to − 2) or IEAC conditioning regimen [substitution of BCNU with idarubicin (8 mg/m^2^ on days − 9 to − 7)]. More than 2 × 10^6^/kg of CD34 + cells were infused into the patients after conditioning regimen. There’s no significant difference of the number of CD34 + cells infused between the two groups. We used ursodiol, acyclovir, SMZ, fluconazole and moxifloxacin as supportive care. No patients received consolidation radiation therapy after transplantation. Rituximab (375 mg/m^2^, every three months) was given to all DLBCL and MCL patients as maintenance therapy for two years.

### Study endpoints

The follow-up deadline was 01 October 2019. The primary endpoint of this analysis was overall survival (OS) among the different conditioning regimens. Secondary endpoints included transplant-related mortality (TRM), relapse or progression, and progression-free survival (PFS). According to WHO criteria, the therapeutic evaluation was divided into complete response (CR), partial response (PR), stable disease (SD) and progressive disease (PD)^18^. Neutrophil and platelet engraftment were defined as the first 3 consecutive days with absolute neutrophil count > 0.5 × 10^9^/L and untransfused platelet count > 20 × 10^9^/L, respectively. Toxicity was assessed using the National Cancer Institute Common Terminology Criteria, version 4.0.

### Statistical analysis

Statistical analysis was performed using SPSS. OS was calculated from the date of diagnosis until death, or until the last follow up date the patient was known to be alive. PFS was determined for responders from the time of diagnosis until disease progression, relapse, death, or until last follow up. PFS was only determined in those complete responders post transplantation. TRM was defined as any death without recurrent lymphoma. The significance of difference between survival curves was calculated by the log-rank test. Groupwise comparisons of the distributions of variables were performed with the generalized Wilcoxon test. A multivariate Cox proportional hazard model with hierarchical forward entering was constructed to assess prognostic factors. Survival and hazard ratio (HR) probabilities were presented with 95% confidence intervals (CI). A *P* value < 0.05 was considered significant different.

### Ethics approval and consent to participate

This study was subject to approval by the Research Ethics Committee of Tianjin Medical University Cancer Institute and Hospital.

## Results

### Clinical characteristics

Patients’ clinical characteristics were shown in Table 1. Of the 72 patients’ retrospective cohort, the median age was 39.5 years old (from 28 to 60 years old), the male to female ratio was 1.32:1. Based on the IPI score, patients were divided into the 0 –2 points group (n = 50, 69.4%) and the 3–5 points group (n = 22, 30.6%). According to Ann Arbor staging system, 33 (45.8%) patients were stage I–II and 39 (54.2%) patients were stage III–IV. There were no significant differences in patient characteristics between IEAC and BEAC groups (Table 1). The pathological type of the 72 newly diagnosed patients was 65 DLBCL cases including nine cases transformed from follicular lymphoma (FL), and 7 MCL cases. All of the patients were primary high-risk lymphoma achieved CR or PR after 4 or 6 cycles of chemotherapy, excluding relapsed disease. After 4 cycles, patients who reached CR (n = 44 for DLBCL, n = 2 for MCL) entered autologous transplantation, while patients who did not reach CR (n = 21 for DLBCL, n = 5 for MCL) continued treatment for 2 cycles, and then entered autologous transplantation. Thirty-five DLBCL patients including 24 in CR and 11 in PR before transplantation were given IEAC conditioning regimen and 30 DLBCL patients (17 in CR and 13 in PR) received BEAC regimen. A total of 5 MCL cases including 3 cases in CR and 2 cases in PR before transplantation were given IEAC regimen. Only two MCL patients received BEAC regimen, both of them were in CR before ASCT.

**Table 1:** Patients’ baseline demographics and clinical characteristics.

	IEAC (n = 40)	BEAC (n = 32)	*P* value
**Gender**			
Male	19 (48%)	22 (69%)	0.10
Female	21 (52%)	10 (31%)	
**Age**			
< 40	22 (55%)	18 (56%)	0.64
≥ 40	18 (45%)	14 (44%)	
**IPI score**			
0–2	27 (67%)	23 (72%)	0.80
3–5	13 (33%)	9 (28%)	
**Ann Arbor stage**			
I–II	20 (50%)	13 (41%)	0.48
III–IV	20 (50%)	19 (59%)	
**LDH level**			
≤ 250	32 (80%)	23 (72%)	0.58
> 250	8 (20%)	9 (28%)	
**Status before ASCT**			
CR	27 (67%)	19 (59%)	0.62
PR	13 (33%)	13 (41%)	
**Pathological type**			
DLBCL	35 (87.5%)	30 (93.75%)	0.42
MCL	5 (12.5%)	2 (6.25%)	

*IPI* international prognostic index, *LDH* lactate dehydrogenase, *CR* complete remission, *PR* partial remission, *ASCT* autologous stem cell transplantation.

### Haematopoietic engraftment

All patients achieved completed haematopoietic engraftment. The median time to neutrophil engraftment (> 500/mm^3^) showed no significant difference between IEAC and BEAC (11 vs 12 days, *P* = 0.23) groups. The median time of engraftment of platelets were 19.5 days (range 13–35 days) in IEAC group and 20 days (range 13–32 days) in BEAC group, still showing no difference (*P* = 0.53, Table 2).

**Table 2:** Hematopoietic engraftment after ASCT.

	IEAC	BEAC	*P* value
	Median (range)	Median (range)	
Time to neutrophils > 500 × 10^3^/mm^3^ (days)	11.0 (9–27)	12.0 (8–24)	0.23
Time to platelets > 20,000 × 10^3^/mm^3^ (days)	19.5 (13–35)	20.0 (13–32)	0.53

### Adverse events

The toxicities between IEAC and BEAC groups were shown in Table 3. We collected the toxicity to day + 100. The most common related adverse events (AEs) observed in all patients were febrile neutropenia (grade 3–4, 70.8%), nausea and vomiting (grade 3–4, 48.6%), oral mucositis (grade 3–4, 11%), cardiac toxicity (grade 1–2, 6.9%), veno-occlusive disease/sinusoidal obstruction syndrome (VOD/SOS, grade 1–2, 4.2%) and central nervus system (CNS) adverse reactions (grade 1–2, 4.2%). IEAC group seemed to have more febrile neutropenia (77.5%) compared to BEAC group (62.5%), however no significant difference was shown between the two groups (*P* = 0.19). No other statistically significant extrahematological toxicities emerged [mucositis (12.5% vs 9.4%, *P* = 0.72), nausea/vomiting (50% vs 46.9%, *P* = 0.82)]. The same situation was observed in VOD/SOS and CNS reaction such as headache (1 patient), convulsion (one patient) and visual impairment (one patient).

**Table 3:** Toxicities between IEAC and BEAC groups.

	IEAC (n = 40)	BEAC (n = 32)	*P* value
Mucositisa	5 (12.5%)	3 (9.4%)	0.73
Febrile neutropenia	31 (77.5%)	20 (62.5%)	0.19
Nausea/vomiting	20 (50%)	15 (46.9%)	0.82
Cardiac toxicity	3 (7.5%)	2 (6.3%)	1.00
VOD/SOS	1 (2.5%)	2 (6.3%)	0.58
CNS reactions	2 (5%)	1 (3.1%)	1.00

*VOD/SOS* Hepatic Veno-occlusive disease (VOD) or Sinusoidal Obstruction Syndrome (SOS), *CNS* central nervous system.

Mucositisa and Nausea/vomiting: grade III.

Cardiac toxicity is the most common side effect of anthracycline drugs. CHOP regimen was the mostly used regimen before transplantation, followed by DA-EPOCH, both of which had doxorubicin. The patients usually received 4 or 6 cycles of chemotherapy pre transplantation, so the cumulative dose of anthracyclines was calculated and within safe doses (≤ 300 mg/m^2^). All of the patients who received anthracycline drugs do ECG and echocardiography every three months. If the patients had heart disease such as heart failure or decreased left ventricular ejection fraction before induction therapy, anthracycline drugs will not be given to the patients, no matter the patients were over 60 years old or not. No one developed cardiac side effects after anthracycline-based induction chemotherapy. All patients assessed the cardiac function before transplantation. Our results showed that addition of idarubicin didn’t increase cardiac issues compared to control group. Due to the short follow-up, it was not adequate to state that cardiac toxicity risk was not increased with an anthracycline-based preparative regimen in long term.

Some patients had liver toxicity or gastrointestinal reaction which can reach grade ≥ 3. Overall severe AEs (by definition of grade ≥ 3) did not differ between these two groups. There was no transplant related mortality (TRM) for all patients indicating that IEAC conditioning regimen was well tolerated.

### Survival analysis

A total of 12 (12/72, 16.7%) patients died due to disease progression. And nineteen patients went into CR from PR after transplantation. For patients who were not in CR after transplantation, we censored them as only CR followed by PFS. The follow-up was restaging after transplantation standard for groups.

**Figure 2:**
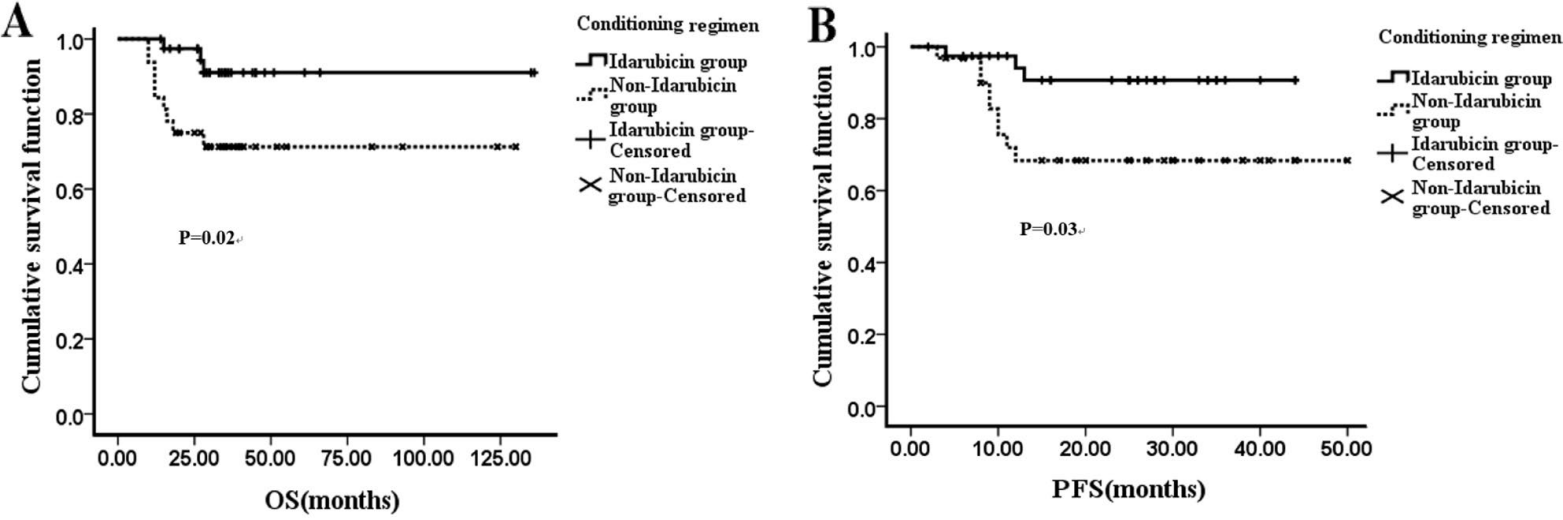
OS and PFS after high-dose chemotherapy followed by ASCT conditioned with IEAC or BEAC. (**A**) The OS of IEAC and BEAC group. (**B**) The PFS of IEAC and BEAC group.

**Figure 3:**
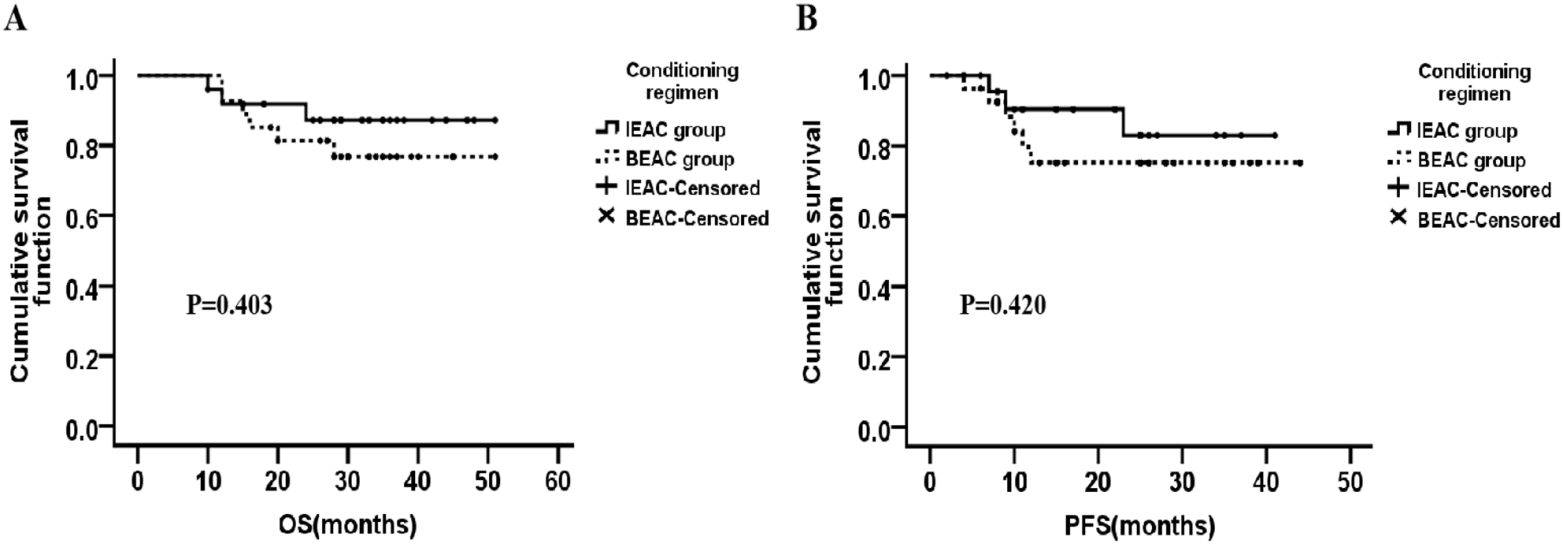
There’s no significant difference in OS and PFS of DLBCL patients between IEAC group and BEAC group.

**Figure 4:**
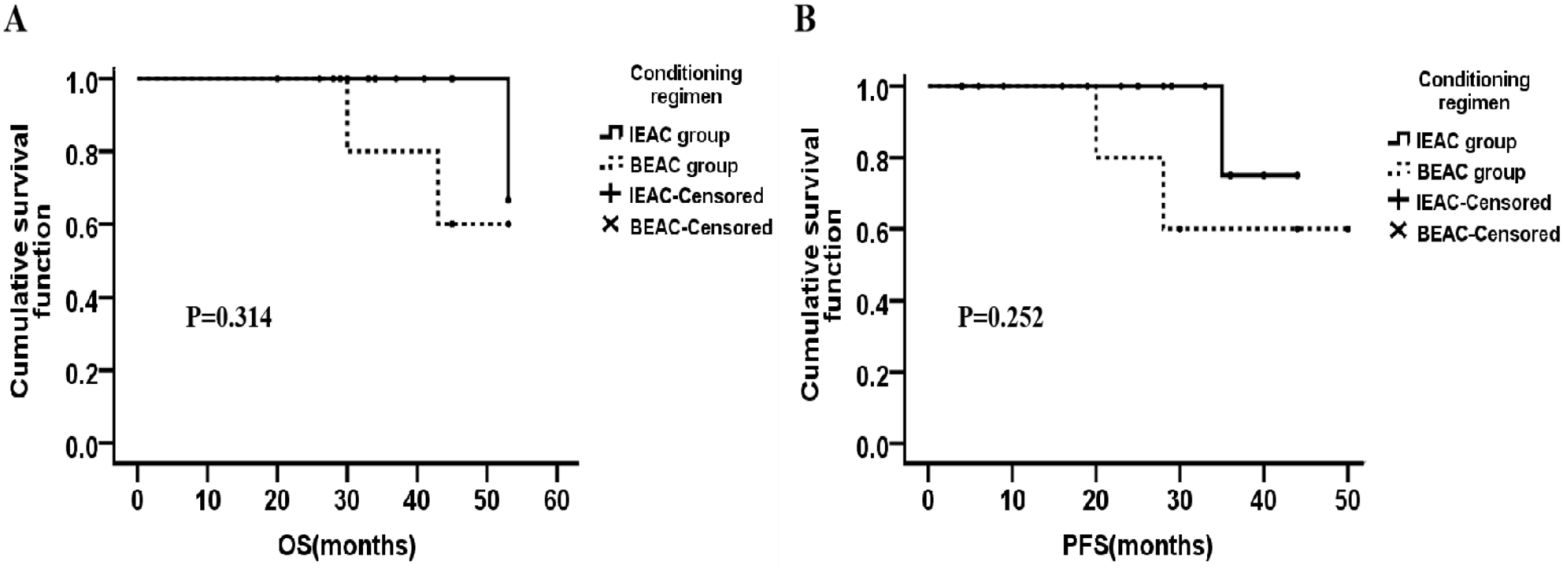
There’s no significant difference in OS and PFS of MCL patients between IEAC group and BEAC group.

The median follow-up time was 31 months. The median OS of IEAC group was 33.0 months [95% confidence interval (CI) 28.50–36.00 months], which was a little longer than that of BEAC group (30.0 months, 95% CI 23.51–35.00 months) (*P* = 0.02, Fig. 2A). Also, the median PFS between IEAC and BEAC groups were 23.0 months (95% CI 16.00–25.50 months) and 18.0 months (95% CI 10.50–27.00 months) respectively (*P* = 0.03, Fig. 2B), indicating that IEAC conditioning regimen may result in better outcomes compared to BEAC. No matter DLBCL or MCL, the prognosis of IEAC groups seemed to be better than that of BEAC groups (Figs. 3, 4), however the *P* values showed no significant difference, maybe due to too few samples.

### Prognosis factors

The univariate and multivariate analysis showed that lactate dehydrogenase (LDH), remission status before ASCT, AnnArbor stage, IPI score and conditioning regimens were prognostic factors relating to OS and PFS. Patients with lower LDH, AnnArbor Stage and IPI score had better prognosis (*P* < 0.05), and patients achieved CR before ASCT had longer PFS (*P* = 0.043) and OS (*P* = 0.045) compared to patients with PR before ASCT. In addition, patients received IEAC conditioning regimen had longer PFS (*P* = 0.02) and OS (*P* = 0.03) than patients in BEAC group (Table 4).

**Table 4:** Univariate and multivariate analysis of factors potentially associated with survivals.

Factors	Univariate				Multivariate			
	OS		PFS		OS		PFS	
	HR (95% CI)	*P* value	HR (95% CI)	*P* value	HR (95% CI)	*P* value	HR (95% CI)	*P* value
**Gender**								
Male (41)	1	0.080	1	0.083	–		–	–
Female (31)	0.342 (0.103–1.137)		0.346 (0.104–1.150)					
**Age**								
< 40 (40)	1	0.888	1	0.806	–		–	–
≥ 40 (32)	0.921 (0.292–2.905)		0.866 (0.274–2.733)					
**IPI score**								
0–2 (50)	1	0.002	1	0.001	–		–	–
3–5 (22)	8.339 (2.251–30.893)		9.350 (2.505–34.899)					
**AnnArbor stage**								
I–II (33)	1	0.049	1	0.061	–		–	–
III–IV (39)	4.591 (1.005–20.969)		4.272 (0.934–19.530)					
**LDH level**								
≤ 250 (55)	1	< 0.001	1	< 0.001	1	< 0.001	1	< 0.001
≥ 250 (17)	0.073 (0.020–0.273)		0.062 (0.016–0.234)		0.072 (0.019–0.269)		0.065 (0.017–0.245)	
**Status before ASCT**								
CR (46)	1	0.045	1	0.043	–	–	–	–
PR (26)	3.428 (1.031–11.402)		3.460 (1.041–11.503)					
**Conditioning regimen**								
IEAC (40)	1	0.023	1	0.034	1	0.041	1	0.032
BEAC (32)	3.494 (0.751–10.284)		3.216 (0.666–10.372)		3.546 (0.757–10.569)		3.843 (0.550–10.172)	
**Pathological type**								
DLBCL (52)	1	0.633	1	0.638	–	–	–	–
MCL (20)	1.375 (0.371–5.091)		1.370 (0.369–5.085)					

*IPI* international prognostic index, *LDH* lactate dehydrogenase, *CR* complete remission, *PR* partial remission, *ASCT* autologous stem cell transplantation.

## Discussion

HDC followed by ASCT could make patients to achieve deeper response, as a result some of them were cured. The PARMA study was the first randomized trial to demonstrate that the use of HDC followed by ASCT resulted in better prognosis compared to standard chemotherapy in patients with relapsed NHL^19–23^. Several studies demonstrated that HDC followed by ASCT as consolidation therapy for patients achieved CR after induction therapy could prolong the PFS, but not the OS^24–26^. Composed drugs of conditioning regimen usually not employed in front-line therapy and not causing high toxicities, BEAC is generally very effective and well tolerated^15,16,27–30^.

Anthracyclines drug such as doxorubicin was commonly used to treat NHL patients. Some studies found that idarubicin was an important anthracyclines drug in lymphoma chemotherapy. Combination of idarubicin and other chemodrugs were utilized as the salvage treatment to achieve high response rate^19,20^. However, few reports demonstrated the efficacy and toxicities of conditioning regimen including idarubicin. Due to the shortage of carmustine, bendamustine and nimustine in China, we modified BEAC protocol by replacing BCNU with idarubicin and examine and evaluate its efficacy and side effects in our single center.

Our results showed that IEAC scheme was well tolerated. As expected, the most frequently observed hematologic toxicity was febrile neutropenia (70.8%), higher than other reports^30–32^, however the median time of neutrophils engraftment did not differ significantly between the IEAC and the BEAC groups. No patient experienced grade IV nausea and vomiting; grade III nausea and vomiting were observed in 50% of patients, higher than other reports. No patient showed significant liver or kidney toxicity and no patient died due to TRM.

The incidence of cardiotoxicity, defined as clinical congestive heart failure (CHF), characterised by pulmonary oedema, fluid overload, and effort intolerance, was dose-dependent with a cumulative doxorubicin^33–35^. Sub-clinical cardiotoxicity is commonly defined on cardiac imaging as clinically asymptomatic left ventricular systolic dysfunction (LVSD) with a fall in left ventricular (LV) ejection fraction (EF) by > 10% points to a value of EF < 50%^36^. The time course of cardiotoxicity varies depending on patient age at time of exposure, the class effect of chemotherapy drugs, and co-existing cardiac risk factors such as hypertension. For all the patients enrolled, the incidence rate of CHF was only 6.9%, much lower than reported previously. Long term monitor showed that no one had LV EF which maybe related to lower heart risk factors such as hypertension. However the median follow-up in the study was 31 months, patients who received idarubicin based preparative regimen after receiving R-CHOP chemotherapy, could be at higher risk of developing long-term toxicities like congestive heart failure with longer follow-up.

For patients with NHL, IEAC produced longer PFS and OS to contemporary patients treated with BEAC, indicating superior outcomes for IEAC. Our results showed that AnnArbor stage, IPI score, LDH level, the remission status before ASCT and conditioning regimen were prognostic factors. Although it was a retrospective study with small case number, and included various histologic types of lymphomas, it was still possible to make some assessments of the efficacy of IEAC. When Carmustine is not available, IEAC regimen could be used as an alternative. However, further prospective, randomized comparative clinical trials should be performed to confirm that IEAC is superior than BEAC.

## Conclusion

In conclusion, IEAC could be used as an alternative while didn’t increase the incidence of toxicities and prolong the median time of hematopoietic engraftment. IEAC has been proven to be safe and effective in different histologic types of lymphoma and, therefore, it may be put forward for consideration.

Due to the retrospective nature and the small sample size, it was difficult to detect survival benefit between these two regimens. However the toxicity profile was similar between IEAC and BEAC with no delay in engraftment, substituting idarubicin especially when there is shortage of carmustine was feasible. Based on the comparable toxicity profile and transplant outcomes, it’s worth evaluating outcomes with IEAC in larger studies.

## Data Availability

The data sets used and/or analyzed during the current study are available from the corresponding author on reasonable request.

## References

[1] Siegel RL, Miller KD, Jemal A (2019). Cancer statistics, 2019. CA Cancer J. Clin..

[2] Ardeshna KM (2005). Conventional second-line salvage chemotherapy regimens are not warranted in patients with malignant lymphomas who have progressive disease after first-line salvage therapy regimens. Br. J. Haematol..

[3] Ayers EC (2020). Outcomes in patients with aggressive B-cell non-Hodgkin lymphoma after intensive frontline treatment failure. Cancer.

[4] Seshadri T, Kuruvilla J, Crump M, Keating A (2008). Salvage therapy for relapsed/refractory diffuse large B cell lymphoma. Biol. Blood Marrow Transplant..

[5] Philip T (1995). Autologous bone marrow transplantation as compared with salvage chemotherapy in relapses of chemotherapy-sensitive non-Hodgkin's lymphoma. N. Engl. J. Med..

[6] Gunnellini M, Emili R, Coaccioli S, Marina LA (2012). The role of autologous stem cell transplantation in the treatment of diffuse large B-cell lymphoma. Adv. Hematol..

[7] Greb A, Bohlius J, Schiefer D, Schwarzer G, Schulz H, Engert A (2008). High-dose chemotherapy with autologous stem cell transplantation in the first line treatment of aggressive non-Hodgkin lymphoma (NHL) in adults. Cochrane Database Syst. Rev..

[8] Chen YB (2015). Impact of conditioning regimen on outcomes for patients with lymphoma undergoing high-dose therapy with autologous hematopoietic cell transplantation. Biol. Blood Marrow Transplant..

[9] Olivieri J (2018). A comparison of the conditioning regimens BEAM and FEAM for autologous hematopoietics stem cell transplantation in lymphoma: an observational study on 1038 patients from fondazione Italiana Linfomi. Biol. Blood Marrow Transplant..

[10] Villa D, Crump M, Keating A, Panzarella T, Feng B, Kuruvilla J (2013). Outcome of patients with transformed indolent non-Hodgkin lymphoma referred for autologous stem-cell transplantation. Ann. Oncol..

[11] Hyung J (2019). Thiotepa, busulfan, and cyclophosphamide or busulfan, cyclophosphamide, and etoposide high-dose chemotherapy followed by autologous stem cell transplantation for consolidation of primary central nervous system lymphoma. Ann. Hematol..

[12] Damon LE (2009). Immunochemotherapy and autologous stem-cell transplantation for untreated patients with mantle-cell lymphoma: CALGB 59909. J. Clin. Oncol..

[13] Visani G (2014). Bendamustine, etoposide, cytarabine, melphalan, and autologous stem cell rescue produce a 72% 3-year PFS in resistant lymphoma. Blood.

[14] Flowers CR (2016). Efficacy of pharmacokinetics-directed busulfan, cyclophosphamide, and etoposide conditioning and autologous stem cell transplantation for lymphoma: comparison of a multicenter phase II study and CIBMTR outcomes. Biol. Blood Marrow Transplant.

[15] Jo JC (2008). BEAC or BEAM highdose chemotherapy followed by autologous stem cell transplantation in non-Hodgkin’s lymphoma patients: comparative analysis of efficacy and toxicity. Ann. Hematol..

[16] Robinson SP (2018). High-dose therapy with BEAC conditioning compared to BEAM conditioning prior to autologous stem cell transplantation for non-Hodgkin lymphoma: no differences in toxicity or outcome. A matched-control study of the EBMT-Lymphoma Working Party. Bone Marrow Transplant..

[17] Engert A (1997). A phase-II study with idarubicin, ifosfamide and VP-16 (IIVP-16) in patients with refractory or relapsed aggressive and high grade non-Hodgkin’s lymphoma. Leuk. Lymphoma.

[18] Jiang M, Bennani NN, Feldman AL (2017). Lymphoma classification update: B-cell non-Hodgkin lymphomas. Expert Rev. Hematol..

[19] Abali H (2005). IIVP salvage regimen induces high response rates in patients with relapsed lymphoma before autologous stem cell transplantation. Am. J. Clin. Oncol..

[20] Oyan B (2005). Ifosfamide, idarubicin, and etoposide in relapsed/refractory Hodgkin disease or non-Hodgkin lymphoma: a salvage regimen with high response rates before autologous stem cell transplantation. Biol. Blood Marrow Transplant..

[21] Cheson B (2014). Recommendations for initial evaluation, staging and response assessment of Hodgkin and non-Hodgkin lymphoma: the Lugano classification. J. Clin. Oncol..

[22] Philip T (1991). Parma international protocol: pilot study of DHAP followed by involved-field radiotherapy and BEAC with autologous bone marrow transplantation. Blood.

[23] Galieni P (2018). Modified BEAM as conditioning regimen for lymphoma patients undergoing autologous hematopoietic stem cell transplantation. Bone Marrow Transplant..

[24] Haioun C (2000). Survival benefit of high-dose therapy in poor-risk aggressive non-Hodgkin's lymphoma: final analysis of the prospective LNH87–2 protocol-a grouped'Etude des lymphomes de l'Adulte study. J. Clin. Oncol..

[25] Chiappella A (2017). Rituximab-dose-dense chemotherapy with or without high-dose chemotherapy plus autologous stem-cell transplantation in high-risk diffuse large B-cell lymphoma (DLCL04): final results of a multicentre, open-label, randomised,controlled, phase 3 study. Lancet Oncol..

[26] Patrick S (2013). Autologous transplantation as consolidation for aggressive non-Hodgkin's lymphoma. N. Engl. J. Med..

[27] Salar A (2001). Autologous stem cell transplantation for clinically aggressive non-Hodgkin’s lymphoma: the role of preparative regimens. Bone Marrow Transplant..

[28] Shi Y (2017). Comparison of CBV, BEAM and BEAC high-dose chemotherapy followed by autologous hematopoietic stem cell transplantation in non-Hodgkin lymphoma: Efficacy and toxicity. Asia Pac. J. Clin. Oncol..

[29] Geisler CH (2012). Nordic MCL2 trial update: six-year follow-up after intensive immunochemotherapy for untreated mantle cell lymphoma followed by BEAM or BEAC + autologous stem-cell support: still very long survival but late relapses do occur. Br. J. Haematol..

[30] Sakellari I (2019). BEAC (carmustine, etoposide, cytarabine, and cyclophosphamide) in autologous hematopoietic cell transplantation: a safe and effective alternative conditioning regimen for Hodgkin and non-Hodgkin lymphoma. Bone Marrow Transplant..

[31] Kuittinen T (2006). Cardiac effects within 3 months of BEAC high-dose therapy in non-Hodgkin’s lymphoma patients undergoing autologous stem cell transplantation. Eur. J. Haematol..

[32] Derenzini E (2008). Pretransplantation positron emission tomography scan is the main predictor of autologous stem cell transplantation outcome in aggressive B-cell non-Hodgkin lymphoma. Cancer.

[33] Sauter CS (2015). Prognostic value of FDG-PET prior to autologous stem cell transplantation for relapsed and refractory diffuse large B-cell lymphoma. Blood.

[34] Isidori A, Christofides A, Visani G (2016). Novel regimens prior to autologous stem cell transplantation for the management of adults with relapsed/refractory non-Hodgkin lymphoma and Hodgkin lymphoma: alternatives to BEAM conditioning. Leuk. Lymphoma.

[35] Von Hoff DD (1979). Risk factors for doxorubicin-induced congestive heart failure. Ann. Intern. Med..

[36] Hequet O (2004). Subclinical late cardiomyopathy after doxorubicin therapy for lymphoma in adults. J. Clin. Oncol..

